# Association of alkaptonuria and low dose nitisinone therapy with cataract formation in a large cohort of patients

**DOI:** 10.1002/jmd2.12288

**Published:** 2022-04-09

**Authors:** Mohammad S. Z. Ahmad, Mahmoud Ahmed, Milad Khedr, Alfredo Borgia, Andrea Madden, Lakshminarayan R. Ranganath, Stephen Kaye

**Affiliations:** ^1^ Department of Ophthalmology Royal Liverpool University Hospital Liverpool UK; ^2^ Clinical Biochemistry and Metabolic Medicine Royal Liverpool University Hospital Liverpool UK

**Keywords:** alkaptonuria, cataract, homogentisic acid, nitisinone, prevalence, tyrosine

## Abstract

Homogentisic acid (HGA) lowering, disease modifying off‐label nitisinone therapy has been used in the United Kingdom National Alkaptonuria Centre (NAC) since 2012. This study evaluated the serendipitous observation of cataract in a large cohort of patients with the very rare disease alkaptonuria (AKU), over a 5‐year period. Patients with AKU who attended the NAC since 2012. Standard physical examination and ocular assessment, including photographs of the crystalline lens were taken before commencement of nitisinone 2 mg daily and annually over 5 years. Photographs were randomised and graded by two independent observers using the WHO cataract classification. AKU patients who did not receive nitisinone were included as a control group. HGA was measured on acidified 24 h urine (u‐HGA_24_) and HGA and tyrosine in fasting acidified serum samples (sHGA, sTYR) at each visit. Patients without suitable lens images were excluded. Cataract (mean grade 1) was noted at baseline in 47 out of 62 (76%) with a mean (SD) age of 44 (14) years. In nitisinone‐treated patients, there were significant increases in the mean grade of nuclear (0.18, *p* < 0.01) and cortical (0.38, *p* < 0.01) lens opacities over the mean duration of 4.93 years of the study. Worsening of the nuclear cataract and cortical lens opacities by at least 1 grade was noted in 14 out of 46 (30%) and 11 out of 46 (24%) patients, respectively. There is an increased prevalence and progression of cataract in AKU and a possible association of nitisinone with cataract progression.

## 1 INTRODUCTION

Nitisinone has recently become available for the treatment of rare iconic metabolic disease alkaptonuria (AKU). The European Medicines Agency approved nitisinone as the first disease‐modifying therapy in adult AKU in September 2020. Most centres treating AKU patients will have limited experience of nitisinone therapy in AKU. On the other hand, nitisinone has been used off‐label as disease‐modifying therapy in the United Kingdom National Alkaptonuria Centre (NAC) commissioned by the Highly Specialised Services NHS England since 2012, making this reporting of our experience with nitisinone in an adult population timely.^1^, ^2^

AKU (OMIM #203500) is a slowly progressive irreversible multisystem disease with a prevalence of around 1 in 500 000 in most non‐consanguineous populations.^3^ The diagnosis of AKU, an autosomal recessive condition, is often made at birth, but patients are not often followed up after initial diagnosis is made in childhood, and reappear later in life with irreversible morbidity. Mutations in the homogentisate dioxygenase gene loci result in loss of enzyme activity, causing increased appearance of homogentisic acid (HGA).^4^ The deposition of HGA as yellow‐black pigment in the eyes, joint and spine cartilage, tendons and ligaments is known as ochronosis.^5^ Ocular features in AKU include characteristic ochronotic pigmentation in the conjunctiva^6^ and sclera,^7^ with pigmentation typically seen in the equatorial regions of the eyeball exposed to sunlight. UV radiation has been suggested as an explanation for the typical scleral location, similar to other disease processes such as pinguecula, pterygium and Bitot's spots seen in vitamin A deficiency.^8^, ^9^, ^10^ Glaucoma, progressive astigmatism and uveitis have also been reported in AKU.^11^, ^12^

Cataract formation refers to increasing cloudiness of the crystalline lens, associated with a decrease in vision by distorting, scattering or blocking light transmission, thus preventing proper focusing of an image on the retina. Incidence of age‐related cataract, the most prevalent type in adults, increases after 50 years of age.^13^ In the healthy lens, protein is mostly in the soluble form, comprising α‐, β‐ and γ‐crystallins at 32%, 53% and 15%, respectively. Lens proteins like all proteins, are susceptible to denaturation and aggregation.^14^ As a molecular chaperone, *α*‐crystallin binds to misfolded proteins and suppresses their nonspecific aggregation,^15^ thus preventing cataract, as proteins are subjected continuously to various environmental and metabolic stresses over the lifetime. Various causes of cataract have been described such as actinic (UV damage), smoking, diabetes, trauma, familial, corticosteroid, myotonic dystrophy and ocular inflammation including uveitis^16^; further, small molecule metabolites such as carbohydrates (galactose, glucose), amino acids (tyrosine, glutathione) and lipids (globotriaosylceramide), and others have been implicated in causing cataract. The prevalence of cataract in hereditary tyrosinaemia type 1 (HT‐1) treated with nitisinone is reported as 1%.^17^

Nitisinone inhibits p‐hydroxyphenyl‐pyruvate dioxygenase (HPPD) enzyme, thereby decreasing the accumulation of HGA and distal tyrosine pathway metabolites.^18^ Nitisinone has been a life‐saving therapy for HT‐1 since 1991, but the experience of nitisinone therapy in HT‐1 is mostly in children.^19^ The decrease in progression of AKU after nitisinone has been shown to be due to lowering of HGA, leading to a reduction in ochronosis, ultimately resulting in improvement in AKU.^1^, ^18^ Off‐label nitisinone 2 mg oral daily has been used in AKU patients at the NAC in The Royal Liverpool University Hospital (RLUH) since 2012. The focus of the NAC has been to show that the off‐label use of nitisinone was both efficacious and safe. The efficacy of nitisinone 2 mg in AKU used in the NAC has been shown in several publications.^20^, ^21^, ^22^, ^23^ Reports on safety in terms of corneal keratopathy and vitiligo following nitisinone use in the NAC have been published.^24^, ^25^, ^26^ Many patients attending the NAC have completed over 8 years of nitisinone therapy thereby allowing longer‐term effects of the drug to be documented. The current data analysis offers an opportunity to understand more fully the prevalence, development and progression of cataract, both in the untreated patients and those on nitisinone.

## 2 PATIENTS AND METHODS

Eighty‐two patients who attended the RLUH with confirmed AKU, through documented increase in urine homogentisic acid, between 2012 and 2020 were included. Nitisinone 2 mg oral was administered off‐licence under approval from NHS England Highly Specialised Services in 71 of these patients. Patients were not on nitisinone at the first visit to the NAC. Patients then attended annually. Eleven patients attended the NAC since 2012 without receiving nitisinone and constituted a no‐nitisinone control group.

A questionnaire collected information about disease features. A standard physical examination, including height and weight was carried out at every visit. Digital photographs of the nasal and temporal aspects of both eyes as well as of both ears were obtained in the medical photography department, and ochronotic pigment scored as previously described.^27^ Serum and urine samples were collected from 2012. HGA was measured on acidified 24 h urine (u‐HGA_24_) and HGA and tyrosine (sHGA, sTYR) in fasting acidified serum samples at each annual visit as previously described, by tandem mass spectrometry.^28^, ^29^

Nitisinone was commenced on day three of the baseline visit, except for the smaller group of 11 patients, who attended for assessment only. When the NAC was commissioned in 2012, available data then indicated that nitisinone 2 mg daily was safe and effective leading to this dose being used.^18^

## 3 OCULAR ASSESSMENT

Case notes were reviewed retrospectively. Visual acuity was recorded and trained ophthalmic technicians took anterior segment photos of the sclera, cornea and the lens.

## 4 GRADING OF LENS OPACITIES

An observer assessed all ocular images and selected the best images to allow lens grading. The images were then randomised and two independent observers subsequently graded all lens images on two separate occasions. The images were graded using the WHO cataract grading system to determine the presence of nuclear, cortical and posterior subcapsular cataract.^30^ Images that were not gradable (WHO level 9) were not included in the analysis. The two observers were masked to the patient's details.

## 5 STATISTICAL ANALYSIS

Descriptive statistics, univariable and multivariable tests were undertaken and SPSS statistical package were used for data analysis. Intra‐ and inter‐observer agreement was assessed using Cohen's kappa. Wilcoxon ranked and Kruskil Wallis tests were used for nonparametric data analysis. A generalised linear model was used to test for significant associations with the change in lens opacities. *p* < 0.05 was considered significant. Bonferroni correction was made for multiple tests.

## 6 RESULTS

**FIGURE 1:**
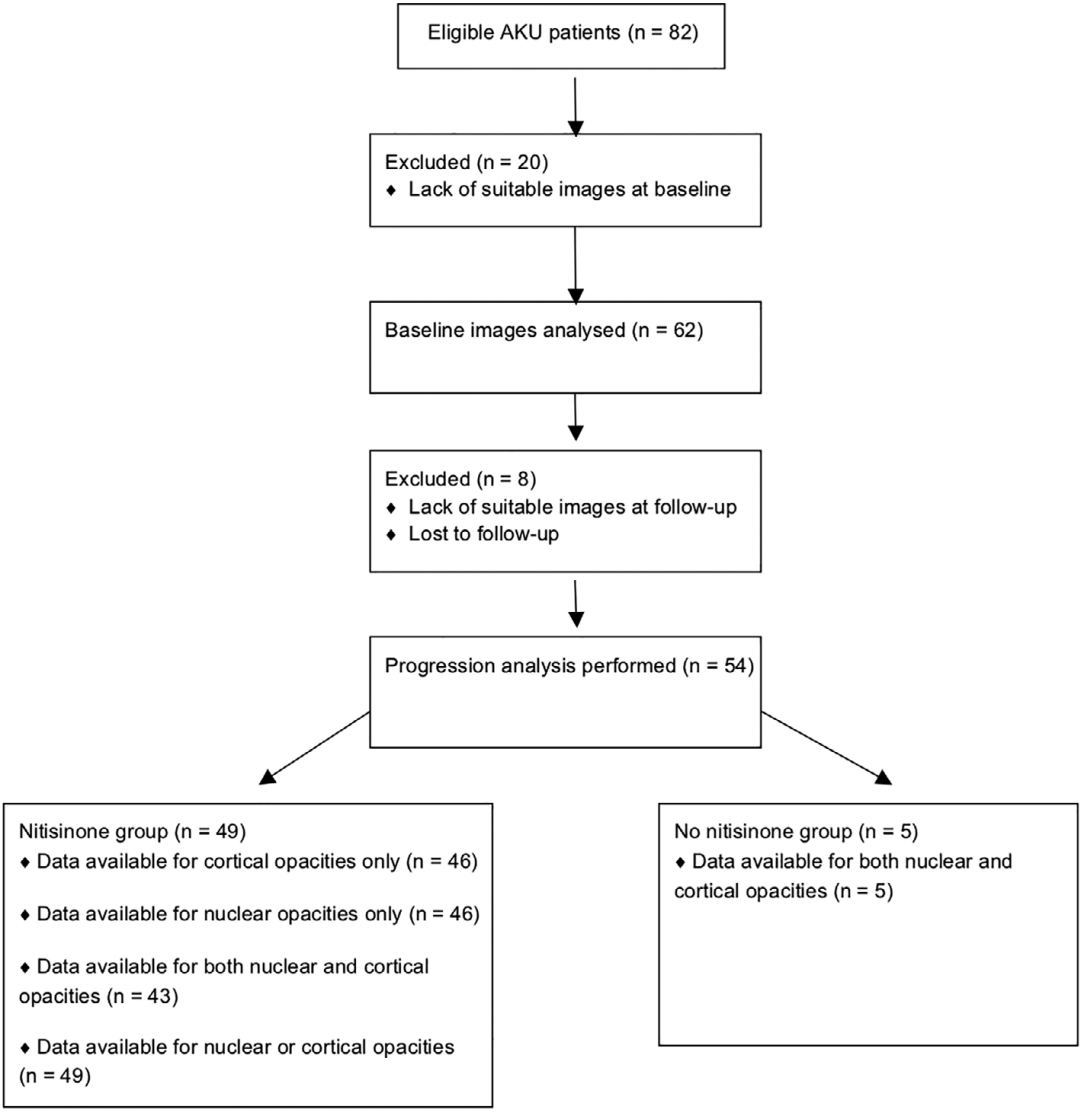
CONSORT flow diagram showing patients that were analysed and excluded

Eighty‐two patients were included comprising 51 males (63%) and 31 females (37%). 20 patients were excluded from the initial analysis of baseline cataract prevalence (*n* = 62) due to a lack of suitable images. A further eight were excluded from progression analysis due to a lack of suitable images or were lost to follow‐up meaning progression analysis was performed on 54 patients, of which five were not on nitisinone and 49 were on nitisinone (Figure 1). 750 images were collected comprising, on average, 12 images per patient (range 4–36) over the course of the study. The image quality was insufficient in 164 images particularly with regards to posterior subcapsular cataract. Therefore, grading was only possible for cortical and nuclear lens opacities. Patient age at the start of study in this group was 44.6 years (16 years to 70 years). The mean (SD) duration of follow‐up was 4.93 (2.1) years. The prevalence of either nuclear or cortical lens opacities at baseline was 47/62 (76%). A summary of the data can be seen in Tables 1, 2, 3. Known contributors to cataract development were only present in a small proportion of patients overall including Diabetes (10%), Smoking (16%), statins (20%) and concurrent steroid use (5%).

**TABLE 1:** Demographic, metabolic, and ocular data in nitisinone‐treated (*n* = 49) and untreated (*n* = 5) AKU patients

	Nitisinone treated (*n* = 49)		No nitisinone (*n* = 5)	
	Mean (SD)	Min to max	Mean (SD)	Min to max
Age at commencement	44.40 (14.28)	16.15–70.42	47.4 (12.5)	30.4–70.4
Age at the end	49.53 (14.27)	19.91–74.41	51.8 (12.6)	31.7–74.4
Change in age	5.13 (1.98)	0.83–8.33	4.38 (1.75)	1.3–7.1
Tyrosine levels at the start μmol/L	68 (62)	20–371	50.9 (13.4)	37–80
Tyrosine levels at the end μmol/L	724 (260)	45–1135	116 (177)	37–649
Change in tyrosine levels μmol/L	656 (256) (*p* < 0.01)	1–1085	65.2–181	−4–608
Cortical opacities start	0.42 (0.58)	0.00–2.00	0.36 (0.67)	0–2
Cortical opacities end	0.76 (0.74)	0.00–3.00	0.64 (0.67)	0–2
Change in cortical opacities	0.38 (0.57) (*p* < 0.01)	0.00–2.00	0.27 (0.47)	0–1
Visual acuity in logMAR at start	0.02 (0.19)	−0.30–1.00	0.1 (0.22)	−0.1–0.5
Visual acuity in logMAR at end	0.05 (0.19)	−0.20–0.80	0.08 (0.19)	−0.1–0.5
Change in visual acuity	0.03 (0.18) (*p* < 0.01)	−0.40–0.70	−0.02 (0.1)	−0.2–0.1
Nuclear opacities at start	0.90 (0.60)	0–2	0.80 (0.45)	0–1
Nuclear opacities at end	1.06 (0.49)	0–2	1 (0)	1–1
Change in nuclear opacities	0.18 (0.39) (*p* < 0.01)	0–1	0.20 (0.45)	0–1
sHGA at baseline (μmol/L)	29 (14.3)	7.6–82.4	25.8 (11.4)	8.1–35.5
uHGA at baseline (μmol/day)	21 782 (8347)	9669–59 310	27 194 (12512)	10 771–45 018

*Note*: *p* values are included for those variables showing a significant change.

**TABLE 2:** Distribution of patients on nitisinone depending on opacity grading at baseline visit and last visit

	Nuclear opacities				Cortical opacities			
	Grade 0	Grade 1	Grade 2	Grade 3	Grade 0	Grade 1	Grade 2	Grade 3
Baseline	11	29	6	0	33	7	6	0
Last visit	4	35	7	0	23	13	8	2

**TABLE 3:** Prevalence and incidence of cataract in the control and nitisinone groups

Feature	No nitisinone group				Nitisinone group			
	Cortical	Nuclear	Cortical or nuclear	Cortical and nuclear	Cortical	Nuclear	Cortical or nuclear	Cortical and nuclear
Prevalence (baseline) %	2/5 (40)	4/5 (80)	5/5 (100)	1/5 (20)	13/46 (28.3)	35/46 (76.1)	35/49 (71.4)	13/43 (30.2)
Prevalence (final visit) %	3/5 (60)	5/5 (100)	5/5 (100)	3/5 (60)	23/46 (50)	42/46 (91.3)	45/49 (91.8)	22/43 (51.2)
Change %	20	20	0	40	21.7	15.2	20.4	21

*Note*: No nitisinone group: *n* = 5; nitisinone group: *n* = 49.

## 7 INTRA AND INTER OBSERVER AGREEMENT IN GRADING CATARACT

The intraobserver agreement (Cohen's kappa) nuclear and cortical lens opacities was 0.51 and 0.53, while the interobserver agreement was 0.47. The agreement between each observer and the lens value recorded in the case records was 0.45 and 0.47.

## 8 CHANGES IN PARAMETERS

The changes in the age, visual acuity, tyrosine and HGA levels, as well as nuclear and cortical opacities in patients who did and did not receive nitisinone are included in Table 1. There was no significant change in visual acuity (0.029 SD 0.18) over the duration of the study (*p* = 0.24) in patients who received nitisinone.

Mean (SD) sTYR increased in patients treated with nitisinone changed from 67.7 (61.8) prior to treatment to 724 (260) μmol/L (*p* < 0.01). In patients who did not receive nitisinone, mean (SD) sTYR was between 50.91 (13.35) and 116.09 (177.43) μmol/L over the duration of the study (*p* = 0.26). The mean (SD) sHGA at baseline in the nitisinone (before nitisinone was administered) and untreated groups were 29 (14.3) and 25.8 (11.4) μmol/L, respectively.

## 9 CHANGE IN LENS OPACITIES

For the patients receiving nitisinone, there were significant increases in the mean grade of nuclear (mean 0.18) and cortical (0.38) lens opacities over the mean duration of 4.93 years in the study (*p* < 0.01, *p* < 0.01) (Table 1). 14 out of 46 (30.4%) and 11 out of 46 (23.9%) patients were noted to have worsening of their nuclear and cortical lens opacities by at least 1 grade, respectively.

For the patients who did not receive nitisinone, there was no significant change in the grade of nuclear (0, SD 0.00) or cortical (0.28 SD 0.47) lens opacities (*p* = 1.00 and *p* = 0.08) over the duration of the study (Table 1). There were no significant associations between the grade of cortical and nuclear lens opacities and the patients age at either the start (*p* = 0.84, *p* = 0.11) or end (*p* = 0.67, *p* = 0.12) of the study. Three out of five (60%) patients had a worsening of their nuclear or cortical lens opacities by 1 grade over the study duration (mean 4.38 years). The changes in nuclear and cortical lens opacities were not related to the patients age (*p* = 0.64, *p* = 0.36) duration (*p* = 0.14, *p* = 0.06), change in visual acuity (*p* = 0.46, *p* = 0.15), or change in sTYR or patients age at either the start or end of the study (*p* = 0.35, *p* = 0.23), respectively.

**FIGURE 2:**
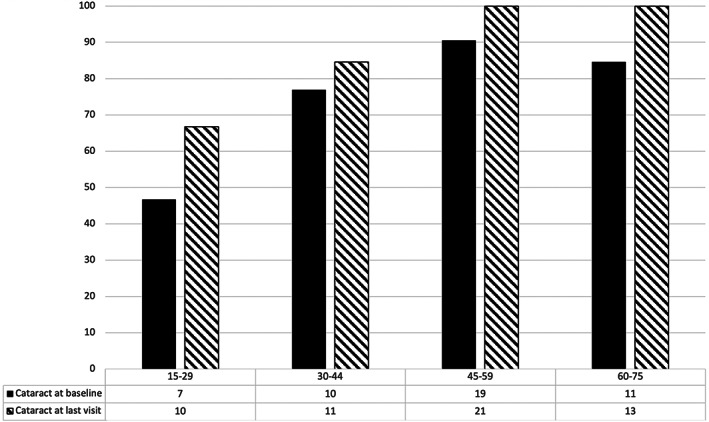
Graph showing the percentage of patients in different age groups with either a nuclear or cortical cataract at beginning and end of the study

Prevalence of any type of cataract at start and end of the study is shown in Figure 2.

## 10 CATARACT SURGERY

Four patients in the cohort, three women and one man, underwent bilateral cataract surgery (Table 4). They were in their sixth to eighth decade of life. Two of the four had cataract surgery while taking nitisinone, whereas the other two started nitisinone after cataract surgery. sHGA and ochronosis scores at baseline ranged from 13.9 to 38.5 μmol/L and 18 to 32, respectively. sTYR ranged widely between visits in each patient.

**TABLE 4:** Data on AKU patients who had cataract surgery

	Patient 1	Patient 2	Patient 3	Patient 4
Current age (y)	74	79	70	67
Sex	M	F	F	F
Age at cataract surgery if known (y)	73	78	Not known	56
Unilateral (U) or bilateral (B)	B	B	B	B
Cataract surgery pre‐nitisinone	No	No	Yes	Yes
Cataract surgery post‐nitisinone	Yes	Yes	No	No
Duration nitisinone therapy (y)	6	6	3	2
sHGA (μmol/L) (baseline)	38.5	31.3	13.9	23
uHGA_24_ (μmol/day) (baseline)	22239	18619	5175	27730
sTYR (μmol/L) (range)	657‐1256	525‐910	432‐1119	626‐983
Ochronosis scores (baseline)	18	27	32	28
Miscellaneous	Vision deterioration at age 71 y	Vision deterioration at age 78		Bilateral cataract surgery age 56 y

## 11 DISCUSSION

The prevalence of cataract in rare metabolic disease AKU is not known. Nitisinone HPPD inhibitor therapy usage in AKU is relatively recent, with proven biochemical efficacy namely the reduction of HGA as well as decreased disease progression.^1^, ^18^ Nitisinone life‐saving therapy in children with HT‐1 has been used since 1991, and at a much higher dose than in AKU.^19^ Experience of using nitisinone in terms of efficacy and safety in adults with AKU is limited. Therefore, more data on safety in nitisinone usage in AKU is needed. To the best of our knowledge, this is the first longitudinal but retrospective study looking at cataract progression in a cohort of patients with AKU receiving nitisinone. Careful ocular assessments were undertaken mainly to detect corneal tyrosine dendritiform keratopathy, which occurred in three NAC AKU patients while on nitisinone.^25^, ^26^ Serendipitous observation of increased prevalence of cataract was observed and as such two blinded independent observers undertook an assessment of the lens opacities in photographs.

Global prevalence of cataract varies by region and a recent meta‐analysis reported that the age‐standardised pooled prevalence estimate of any cataract was 17.2% and estimated the prevalence of any cataract in people increased with age in 3% of 20–39 year olds, 17% of 40–59 year olds and as high as 54% in people over 60 years.^31^ The prevalence of cortical cataracts in these age groups was reported as 2%, 7% and 25%, respectively, while nuclear cataract was described in 1%, 6% and 31%, respectively. A UK study estimated the prevalence of cataract causing visual impairment in those over 65 years old at around 30%.^32^ In contrast, the overall prevalence of cataract in our patient cohort with a mean age of around 44 years was 76% at baseline. This was considerably higher in each of the age groups than the corresponding general population age groups in the above‐mentioned studies. This would suggest a much higher presence of both cortical and nuclear cataracts in patients with untreated AKU. We also noted a high prevalence of cataract in young patient groups including between 15 and 30 years as well as between 30 and 45 years, which appears to be much higher than expected for this age range (Figure 2).

The increased cataract prevalence in untreated AKU could be related to lifelong increase and exposure to HGA. sHGA has also been associated with an increase with age.^33^ The formation of tissue altering HGA‐ochronotic pigment is the main mechanism by which HGA causes damage and disease in AKU. Ochronosis in AKU is mediated by HGA‐induced oxidative mechanisms.^34^ HGA undergoes spontaneous oxidation into 1,4‐benzoquinone‐2‐acetic acid (BQA), a process that can also lead to production of oxygen radicals such as superoxide anion, hydroxyl radical and hydrogen peroxide.^35^ Therefore, HGA in the ocular tissues including in the lens can be associated with oxidative stress since birth. The usual age‐related cataract is also caused by UV oxidant‐generated free radial stress, which depletes the protective α‐crystallin and thereby causes the aggregation of the βγ‐crystallins the precursor of lens opacification. Moreover, there is evidence in the literature that HGA induces aggregation and fibrillation of amyloidogenic proteins, which might also contribute to cataract formation.^36^ It has been suggested that ochronotic pigment can accumulate in the human lens although we were not able to confirm its presence in our dataset.^12^ The proposed mechanism of HGA‐mediated oxidant stress in AKU leading to cataract may be similar to diabetes where accelerated cataract formation occurs, and where the free radical superoxide in the mitochondria has been shown to be elevated as a result of hyperglycemia.^37^ Moreover, glutathione, or γ‐glutamyl‐cysteinyl‐glycine, is a tripeptide present in high concentration in the lens where it functions as an essential anti‐oxidant for maintenance of lens transparency.^38^ Glutathione deficiency in diabetes is thought to contribute to oxidative stress and microvascular complications.^39^ Similarly in AKU, glutathione is reduced with increase in HGA prenitisinone treatment^40^ and may also be reduced with increase in tyrosine during nitisinone therapy.^41^ The depletion of glutathione is a decrease in one of the important anti‐oxidant defences thereby increasing oxidant stress and in turn contribute to cataract formation. Moreover, AKU patients struggle with lower‐protein diets throughout their life and dietary factors influencing cataract formation cannot be excluded in these patients.^42^ A recent analysis of the diet in the NAC patients showed significant malnutrition including sarcopenia even before nitisinone therapy and a nutrition deficiency contribution to cataract in AKU cannot be excluded.^42^, ^43^

When assessing cataract progression, our findings suggest that progression of cataract formation is higher in patients with AKU than in the general population. One study of an Icelandic population above the age of 69 years reported a progression rate over 5 years of 39% for nuclear and 22% for cortical cataracts.^44^ The progression rates in our study were for a very much younger age group and were 15–20% for nuclear and cortical cataracts.

Further, in the NAC analysis described here, cataract progression was significant only in those on nitisinone with the limitation that the numbers of untreated AKU patients who were followed up were smaller. It is therefore possible that nitisinone may have caused cataract development. The inevitable consequence of nitisinone therapy is universal tyrosinaemia, and this was also observed in the NAC nitisinone‐treated patients. sTYR increased from low normal baseline values more than 10‐fold during nitisinone therapy. Tyrosine concentrations have been shown to be around five‐times higher in the lens compared with the aqueous humour which in turn is double that of circulating tyrosine.^45^ A full range of amino acids are needed for the synthesis of lens proteins, and tyrosinaemia can cause an imbalance in lens amino acid concentrations due to competition with other amino acids for the LATS‐1 transporter.^46^ Tyrosine is involved in redox reactions like HGA and could cause an oxidant stress in the lens.^47^ Association of tyrosine and cataract formation has been described previously in animal studies by Srivastava and Beutler who proposed that presence of tyrosine in the lens can lead excess production of dopaquinone, which could then combine with lens proteins and may cause cataract formation.^48^ It is not currently known whether nitisinone‐induced tyrosineamia compounds the lifelong exposure of the lens to HGA, with both HGA and TYR having pro‐oxidant properties. The data in this study provides some support for effect of nitisinone on cataract progression, as there was a further increase in cataract despite decrease in HGA. This is important to note as it may also implicate tyrosine in the cataract process. This question is important since nitisinone‐associated tyrosinaemia is also present in HT‐1 but in which the prevalence of cataract has been reported as 1%.^17^

Although there was no significant deterioration in visual acuity during the course of the study, it is possible that with longer and more appropriate follow‐up this may change. In addition, development of cataract is not always associated with a proportional, measurable deterioration in visual acuity. There were four patients in their sixth and eighth decade who underwent cataract surgery due to a deterioration in visual acuity. The metabolic data in these patients undergoing surgery appeared to be similar to the overall group data.

Our data suggests that both lifelong exposure to HGA and tyrosinaemia during nitisinone therapy may be involved in the increased risk of cataract in the NAC cohort. AKU is a serious severe debilitative multisystem disorder for which nitisinone is the only approved therapy. In this context, since cataract prevalence is greatly increased in untreated AKU with a further smaller increase after nitisinone therapy, it may be possible to justify nitisinone therapy. Nitisinone has beneficial effects in other tissues and organs and may counterbalance the increased risk of cataract during nitisinone therapy. A greater understanding of the mechanisms in untreated and nitisinone‐treated AKU is needed to develop strategies to prevent cataract formation.

### 11.1 Limitations

The above analysis appears to suggest a much higher prevalence of cataract at baseline in our study population compared with age‐matched healthy population reports in the literature. Although the population size is limited in the current study, AKU is an ultra‐rare disease and large patient numbers would not be expected. Our findings would suggest that AKU could be associated with an earlier onset of cataract, an important finding as currently cataract is not widely known to be associated with AKU.^49^ We were only able to find three reports in the literature of cataract reported in patients with AKU, some of which were congenital cataracts.^50^, ^51^

Although worsening of lens opacity in this study did not affect visual acuity, we cannot exclude early visual impairment as contrast acuity, colour vision or other parameters of vision were not measured. Measures were taken to minimise bias in the study. The case notes and clinical photos of the patients were reviewed and collected by an independent ophthalmologist. Thereafter, the collected images were anonymised and graded by two independent observers on two separate occasions who were not aware of the increased prevalence of cataracts in this cohort. Levels of agreement were moderate. A significant proportion of images were excluded for technical reasons and it is unclear what effect these might have had on the overall results and conclusions. A further limitation of the study is that there was no healthy control group without AKU and the number of patients who did not receive nitisinone was small; however, the present analysis was carried out as an audit of a protocolised service and not as a research study and the results on lens opacities was unexpected and unanticipated. Moreover, there were no appropriate images in this study to determine the presence of posterior subcapsular cataract. Moreover, this study only reports data over a time period of around 5 years, and it is possible that clearer trends may become apparent if longer‐term follow‐up data were available. In conclusion, despite the small sample size, this study appears to show an association between untreated AKU and cataract development, possibly due to HGA, for the first time. Progression of cataract during nitisinone therapy was observed over a relatively short time period across all ages including in the younger patients implicating tyrosinaemia as a mechanism. We suggest that the high lifetime exposure to HGA compounded by tyrosinaemia during nitisinone therapy contributed to the development of lens opacities. Further studies of better quality and for longer duration are required to clarify these findings.

## CONFLICT OF INTEREST

The authors declare no conflict of interest.

## AUTHOR CONTRIBUTIONS

Mohammad S. Z. Ahmad: Data collection, analysis, manuscript drafting. Mahmoud Ahmed: data collection, analysis, manuscript drafting. Milad Khedr: assessed patients in the National Alkaptonuria Centre. Alfredo Borgia: Data collection, analysis, manuscript drafting. Andrea Madden: ocular assessment and obtaining lens photographs. Lakshminarayan R. Ranganath: conceived this project and edited and drafted the manuscript. Stephen B. Kaye: manuscript idea and planning, analysis, manuscript drafting.

## ETHICS APPROVAL

Data from the United Kingdom National Health Service Highly Specialised Services approved service, the National Alkaptonuria Centre (NAC), was collected and analysed as part of annual institutional audit (Audit no. ACO3836). A booklet describing all processes carried out in the NAC was given to each participant before they agreed to come to the NAC; specific information was provided in the booklet that data collected would be published but subjects would not be identifiable during the dissemination process. Further, specific consent was obtained for the ear and eye photographs using the institutional consent mechanisms. Data from the NAC has been published in peer‐reviewed journals previously along similar justifications. The highest standards of ethical and clinical practices were followed in delivering the service at the NAC. All procedures followed were in accordance with the ethical standards of the responsible committee on human experimentation (institutional and national) and with the Helsinki Declaration of 1975, as revised in 2000 (5). Ethical approval or informed consent was not required as this study was carried out as an audit. This article does not contain any studies with human or animal subjects performed by the any of the authors.
